# Exploring interventions to support life participation for adults with chronic kidney disease: a scoping review

**DOI:** 10.1186/s12882-025-04162-8

**Published:** 2025-05-22

**Authors:** Mikaela Correa, Roaa Hussam, Janine Farragher

**Affiliations:** Department of Occupational Science & Occupational Therapy, Temerty Faculty of Medicine, University of Toronto, 160-500 University Ave, Toronto, ON Canada

**Keywords:** Life participation, Chronic kidney disease, Occupational engagement, Quality of life, Disability, Occupational therapy, Rehabilitation, Exercise

## Abstract

**Background:**

Individuals with chronic kidney disease (CKD) can experience significant health-related challenges that affect their life participation. Recent studies have identified life participation as a top priority for adults with CKD. This scoping review aims to comprehensively identify studies of interventions that targeted life participation outcomes in adults with predialysis CKD, and identify gaps in the literature.

**Methods:**

This scoping review followed the Joanna Briggs Institute (JBI) methodology. Five electronic databases were searched with additional sources identified through backwards chaining. Title and abstract screening were conducted independently by four screeners after initial inter-rater calibration, and full text screening and data extraction were undertaken by two researchers in duplicate. Data analysis was completed using descriptive statistics and narrative synthesis.

**Results:**

This scoping review identified twenty-three studies that examined interventions to support life participation in the CKD population. No studies targeted life participation as a primary outcome. When categorizing studies via the Canadian Model of Occupational Performance and Engagement (CMOP-E) the majority (87%) of interventions targeted personal-physical mechanisms of disability, with a predominant focus on pharmacological (48%) or exercise (35%) interventions. Dedicated outcome measures for life participation were rarely used, with the role-physical, role-emotional and social functioning subscales of the SF-36 quality of life assessment being the most common life participation outcome measures.

**Conclusions:**

This scoping review highlights a lack of research and prioritization of life participation in CKD. It demonstrates the narrow scope of intervention approaches used to support life participation, and limitations in how studies assess life participation. These gaps indicate a need for further research to support this top priority health outcome for the CKD community.

**Clinical trial number:**

As this is a scoping review, no clinical trial number is provided.

**Supplementary Information:**

The online version contains supplementary material available at 10.1186/s12882-025-04162-8.

## Background

Approximately 10% of the global population experience chronic kidney disease (CKD) [1]. People with CKD can experience many life changing health complications, which can affect their ability to engage in meaningful life activities such as self care, leisure, and productive activities. CKD has a high burden of disability and is listed among the top 20 leading causes of disability-adjusted life years globally [2]. Research has shown that the presence of CKD is associated with a significant decline in independence while performing instrumental activities of daily living, such as shopping and managing money, and basic activities of daily living, such as toileting, and eating [3]. Studies also show that individuals with CKD experience decreases in social contact, social participation, and community mobility [4, 5]. Life participation, defined as participating in one or more meaningful activity of life, has been shown to be a top priority for adults with chronic kidney disease [6]. Engagement in valued activities brings meaning to life, and it has been said that “sick minds, sick bodies and sick souls may be healed through occupation” [7](p.14). It has also been found that engaging in meaningful activities can be an essential aspect of improving the health of individuals with ongoing health conditions [8]. With such an impact on health and quality of life, it is imperative to identify interventions that can improve the ability to engage in life activities in the CKD population.

There are a number of health challenges associated with CKD that might contribute to its impact on engagement in meaningful activity. CKD has been found to be “ an independent contributor to decline in physical and cognitive functions in older adults’’ [9]. Some of the physical limitations individuals with CKD can experience include muscle wasting, weakness, low levels of physical activity, and frailty [9–11]. The cognitive domains most affected by CKD are orientation, attention and language [12]. Moreover, there are also effects on the mental health of individuals with CKD with many experiencing depression (20–25%), anxiety (19%), anxiety symptoms (49%) and psychological distress (26%) [13–15]. All of these sequalae of CKD have the potential to profoundly impact engagement in meaningful activities.

The need for quality research on interventions to enable life participation in the CKD population has previously been raised in the literature. For example, a 2022 article investigating lifestyle interventions for “enabling people to live well” with CKD highlighted a lack of quality research [16]. The author discusses the most recent international guideline for “the evaluation of chronic kidney disease” and highlighted that although supportive interventions such as receiving expert dietary advice and psychological care are recommended for people with CKD, there is limited evidence to support the claims [16]. The author also highlights how robust intervention recommendations exist for other chronic conditions such as diabetes and multiple sclerosis, but are lacking in chronic kidney disease [16]. Exercise is among the more common rehabilitative interventions studied in the CKD population, however few exercise programs have focused on enabling life participation [17]. Similarly, self-management interventions are relatively common interventions studied among the CKD population, but few have been found to target life participation [18, 19].

We therefore conducted a scoping review to systematically examine and characterize interventions that have been researched to address occupational engagement in adults with chronic kidney disease.

## Methods

This scoping review followed the Joanna Briggs Institute (JBI) methodology for scoping reviews [20]. As per the JBI decision tree for selecting scoping review methodology, the purpose of the review was to identify the types of available evidence, and to identify and analyze knowledge gaps. Our scoping review manuscript was written according to the PRISMA-ScR reporting guidelines for scoping reviews [21].

### Search strategy

As per JBI scoping review guidelines, this scoping review aimed to identify published literature and grey literature [20]. Electronic database searches were conducted using a comprehensive set of search terms representative of the target population (CKD) and concept (life participation). The databases searched include MEDLINE (OVID), EMBASE, PsycINFO, Cochrane Central Register of Controlled trials and CINAHL Plus. A search for grey literature in the form of published abstracts was also conducted via Google Canada and Scopus. The most recent search was completed on December 20, 2023. Additional information sources were identified through backwards chaining using reference list scanning.

### Study/source of evidence selection

This scoping review followed a two-level screening process involving at least two reviewers, as outlined by the JBI method for scoping reviews. Duplicates were removed using the Covidence review management software. Screening questions were developed and used to determine source inclusion eligibility. Identified sources first went through level 1 screening, where titles and abstracts were screened based on the screening questions. Four screeners completed level 2 screening independently after undergoing a piloting and calibration exercise to ensure adequate inter-rater reliability (Kappa > 0.8). Level 2 screening involved full text review of identified sources by two independent screeners in duplicate. Reasons for exclusion of sources during level 2 screening were recorded for transparency. Any disagreements between reviewers were resolved through discussion and if consensus was not attainable, a third member of the research team assisted with reaching consensus.

### Inclusion criteria

Any qualitative, quantitative study or mixed-methods study of an intervention used with the pre-dialysis CKD population that reported its impact on life participation was eligible for inclusion, as per the following criteria:

1. Study population was adults 18 + with predialysis chronic kidney disease (stage 1–4 or EFGR > 15).
2. Study investigated any type of intervention.
3. Study measured life participation as an outcome of the intervention, defined as performance or participation in one or more meaningful activities of life.

Studies meeting the above criteria that were available in English from any year or location were included in the review.

### Data extraction and analysis

Data extraction was completed by two independent researchers in duplicate and stored in a secure Microsoft Excel sheet. Extracted data were then discussed to resolve discrepancies and reach consensus. Data extracted included information on article, intervention, and research characteristics. Interventions used in the literature were classified into descriptive categories based on the Canadian Model of Occupational Performance–Engagement (CMOP-E) domains [22], which describe the range of personal, environmental and occupational factors that can impact life participation, as outlined in Table 1 [22]. Data were analyzed using descriptive statistics and narrative synthesis. Counts and percentages were used to identify patterns in quantitative data for relevant concepts and populations. Narrative synthesis was used to analyze themes present in the qualitative data, and data were presented via graphs and tabular charts.

**Table 1:** CMOP-E domains [22]

CMOP–E	Definition
Personal–Physical	Movement, strength, coordination, balance, endurance, pain, appearance, and physical illness and/or injury of body systems and/or structures
Personal–Cognitive	Memory, orientation, concentration, intellect, problem-solving, insight, judgment, general knowledge
Personal–Affective	Emotions, mood, affect, volition, body image, coping skills, and reaction and adaptation to illness or injury
Occupational	Groups of activities and tasks of everyday life, named, organized, and given value and meaning by individuals and a culture
Environmental–Physical	Built and natural aspects of the environment, from micro (e.g. type of doorknobs) to macro (e.g. how society is built to provide accessible space)
Environmental–Social	People in the environment, our relationships with them, the networks we can leverage when requiring assistance, and the general availability of assistance to support living
Environmental–Cultural	Micro-level, such as person or family rituals, and macro-level, such as expectations and attitudes of the broader culture
Environmental–Policy	Governmental and organizational structures, policies and practices, as well as hidden or under-considered social determinants of health

## Results

**Fig. 1:**
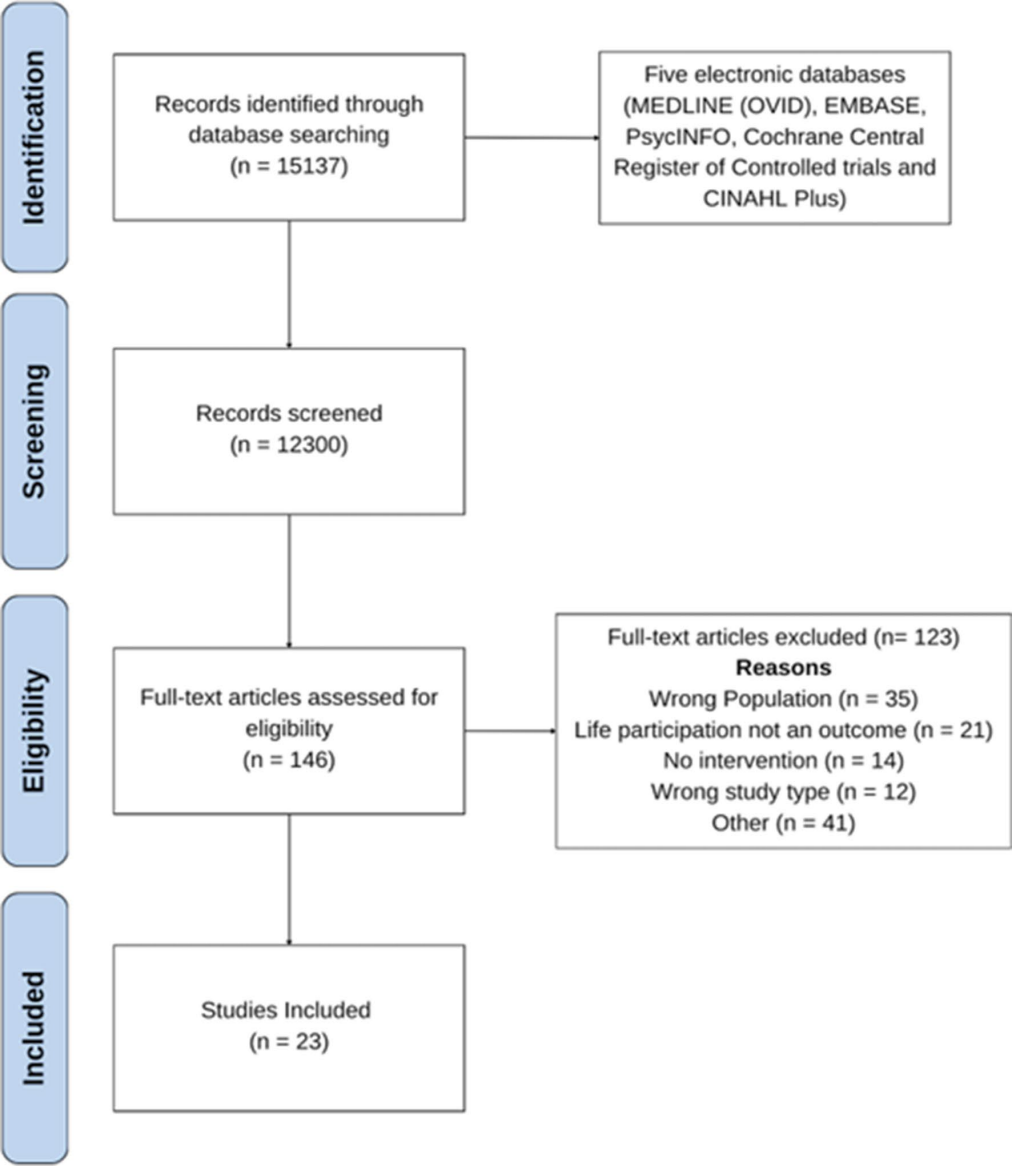
PRISMA flowchart of study selection process

A total of 15,137 records were identified through database searching. After removing duplicates, screening and assessing articles for eligibility, a total of twenty three studies met the inclusion criteria for this scoping review. The PRISMA flowchart detailing the study selection process is shown in Fig. 1.

### Characteristics of included studies

The characteristics of the included studies are detailed in Table 2. Included studies were published between 1995 and 2023. The most common countries of origin were the United States of America (*n = 5*; 22%) and the United Kingdom (*n = 4;* 17%) with other countries including Canada, India, China, Brazil, Sweden, Japan, Australia, France, Taiwan, and the Republic of Korea. Most of the studies were randomized control trials (*n = 18*; 78%) with the other design being pre-post (*n = 3;* 13%) and qualitative (*n = 2;* 9%). Sample sizes of the studies ranged from 9 to 390 with a median sample size of 79.

**Table 2:** Characteristics of included studies

Year	Title	Author	Country	Study design	Sample size	Intervention type	Intervention description	Intervention provider	Life participation focus	Life participation measures used	Reported life participation outcomes
2023	Patient reported outcome measures and cardiovascular outcomes following high dose modern intravenous iron in non-dialysis dependent chronic kidney disease: secondary analysis of ExplorIRON-CKD	Kassianides [28]	UK	RCT	26	Personal – Physical	Participants received either ferric derisomaltose or ferric carboxymaltose intravenously.	Nurse	Secondary	SF-36 - role physical, role emotional, social function; Duke Activity Status Index	Mixed
2022	Effects of A 16-week physical training on mortality, quality of life, and CKD progression: Nephros post-trial follow-up	Bohlke [29]	Brazil	RCT	105	Personal – Physical	Participants engaged in aerobic and resistance training.	Not reported	Secondary	SF-36 - role physical, role emotional, social function	Not improved
2022	Enzobiotics—A Novel Therapy for the Elimination of Uremic Toxins in Patients with CKD (EETOX Study): A Multicenter Double-Blind Randomized Controlled Trial	Saxena [30]	India	RCT	85	Personal – Physical	Participants received interventional proprietary enzobiotic capsules 5 min before each major meal. Participants also followed a CKD diet prescribed by a renal dietician.	Not reported	Secondary	SF-36 - daily activity limitations	Not improved
2022	Perceptions and Experiences of a Progressive Resistance Exercise Program in People with Chronic Kidney Disease	Lightfoot [31]	UK	Qualitative	9	Personal – Physical	Participants engaged in a resistance exercise intervention consisting of leg extensions.	Not reported	Secondary	Qualitative interviews	Improved
2022	Analysis of the Efficacy of Alprostadil in Combination with Reduced Glutathione on the Chronic Renal Failure	Chen [32]	China	RCT	98	Personal – Physical	Participants received alprostadil in combination with reduced glutathione.	Not reported	Secondary	SF-36 - social function	Improved
2022	Experiences of Intravenous Iron Therapy in Non-Anaemic Functional Iron Deficient Individuals With Non-Dialysis Kidney Disease: A Qualitative Study	Lightfoot [33]	UK	Qualitative	17	Personal – Physical	Participants received intravenous iron infusions and participated in exercise training.	Not reported	Secondary	Qualitative interviews	Not improved
2021	Effects of AST-120 on muscle health and quality of life in chronic kidney disease patients: results of RECOVERY study	Cha [34]	Republic of Korea	RCT	150	Personal – Physical	Participants received AST-120 in addition to standard care.	Self administered	Secondary	SF-36 - role physical, role emotional, social function; KDQOL - quality of social interaction, work status, social support	Not improved
2021	Home-based aerobic exercise and resistance training in pre-dialysis patients with advanced CKD: A randomized controlled trial	Uchiyama [35]	Japan	RCT	46	Personal – Physical	Participants engaged in home-based individualized aerobic exercise and resistance training. Resistance training involved using a Theraband to isolate one muscle group at a time.	Rehabilitation doctor & self-supervised	Secondary	SF-36 - role physical, role emotional, social function; KDQOL - quality of social interaction, work status	Mixed
2020	A prospective, double-blind, randomized, placebo-controlled interventional study to evaluate the safety and efficacy of enzobiotics in pre-dialysis CKD patients	Saxena [36]	India	RCT	80	Personal – Physical	Participants received enzobiotic capsules 5 min before food.	Not reported	Secondary	SF-36 - daily activity limitations	Improved
2020	Mobile health app with social media to support self-management for patients with chronic kidney disease: prospective randomized controlled study	Li [37]	Taiwan	RCT	60	Multi-domain (Personal – Physical, Personal – Cognitive, Personal – Affective)	Participants received education on self-management, diet and exercise. Participants had a daily target of 7500 steps and used wearable devices to collect exercise related data. Participants used the LINE mobile app to learn about diet, exercise and CKD management.	Trained staff	Secondary	SF-36 - role physical, role emotional, social function; KDQOL - quality of social interaction, work status; The Self-Management Questionnaire - self-care	Mixed
2019	Long-term safety and efficacy of veverimer in patients with metabolic acidosis in chronic kidney disease: a multicentre, randomised, blinded, placebo-controlled, 40-week extension.	Wesson [38]	USA	RCT	217	Personal – Physical	Participants received veverimer suspended in water with food.	Trained staff	Secondary	KDQOL-PFD - daily activities	Not improved
2019	Twelve weeks of supervised exercise improves self reported symptom burden and fatigue in chronic kidney disease: a secondary analysis of the “ExTra CKD” Trial	Wilkinson [39]	UK	RCT	36	Personal – Physical	Participants in both the aerobic and combined exercise groups engaged in moderate intensity aerobic activity on standard cardiovascular equipment. The combined exercise group engaged in resistance training via leg extension and leg press exercises, for 10 min of their session.	Exercise trainer	Secondary	SF-36 - role physical, role emotional, social function	Mixed
2018	Meeting patients where they are: Improving outcomes in early chronic kidney disease with tailored self-management support (the CKD-SMS study)	Havas [40]	Australia	Pre-post	78	Multi-domain (Personal – Cognitive, Personal – Affective, Environmental – Social)	Participants engaged in goal-setting intervention sessions and phone sessions that utilized techniques from SCT, motivational interviewing, cognitive behavioural and mindfulness techniques. Participants were also given a handbook and handouts on self-monitoring and notetaking.	Principle researcher	Secondary	Human Activity Profile - self-care, personal/housework, entertainment/social	Mixed
2017	Home-based versus center-based aerobic exercise on cardiopulmonary performance, physical function, quality of life and quality of sleep of overweight patients with chronic kidney disease	Aoike [41]	Brazil	RCT	45	Personal – Physical	Participants in the home-based group underwent 3 initial supervised exercise sessions and then walked at locations near their home. Participants in the centre-based group trained on a treadmill at an exercise centre. Participants in both groups followed a renal specific diet.	Exercise physiologist	Secondary	SF-36 - role physical, role emotional, social function	Mixed
2018	Agomelatine versus paroxetine in treating depressive and anxiety symptoms in patients with chronic kidney disease	Chen [42]	China	RCT	108	Personal – Physical	Participants received either paroxetine in the morning or agomelatine before bed.	Not reported	Secondary	Activities of Daily Living Scale	Improved
2017	Vitamin D3 supplementation, bone health and quality of life in adults with diabetes and chronic kidney disease: Results of an open label randomized clinical trial	Mager [43]	Canada	RCT	120	Personal – Physical	Participants received either daily or monthly vitD3.	Not reported	Secondary	SF-36 - role physical, role emotional, social function	Mixed
2014	Effects of a renal rehabilitation exercise program in patients with CKD: a randomized, controlled trial	Rossi [44]	USA	RCT	119	Personal – Physical	Patients participated in group or individual guided exercise sessions on top of standard CKD care. This included cardiovascular, weight training and stretching exercises.	Exercise physiologist or physical therapist and trained staff	Secondary	SF-36 - role physical, role emotional, social function	Mixed
2012	Empowerment intervention in outpatient care of persons with chronic kidney disease pre-dialysis	Nygårdh [45]	Sweden	Pre-post	46	Multi-domain (Personal – Cognitive, Personal – Affective)	Participants engaged in learning meetings that utilize person-controlled education.	Nurse	Secondary	EQ-5D - self care, usual activities	Mixed
2010	Effects of exercise training on physical impairment, arterial stiffness and health-related quality of life in patients with chronic kidney disease: a pilot study	Mustata [46]	Canada	RCT	20	Personal – Physical	Participants engaged in in-centre aerobic sessions and home walking training.	Not reported	Secondary	SF-36 - role physical, role emotional, social function; EQ-5D - self-care, usual activities	Mixed
2009	Predialysis chronic kidney disease: evaluation of quality of life in clinic patients receiving comprehensive anemia care	Hansen [47]	USA	Pre-post	79	Personal – Physical	Participants received anemia therapies and education.	Not reported	Secondary	KDQOL- work status; SF-36 - role physical, role emotional, social function	Mixed
2007	Association of anemia correction with health related quality of life in patients not on dialysis	Alexander [48]	USA	RCT	62	Personal – Physical	Participants received darbepoetin alfa in addition to conservative management of CKD.	Not reported	Secondary	SF-36 - role physical, role emotional, social function; KDQOL - social interaction, ADL/IADL	Mixed
2006	Effect of early correction of anemia on the progression of CKD	Rossert [49]	France	RCT	390	Personal – Physical	Participants received epoetin alfa subcutaneously	Not reported	Secondary	SF-36 - role physical, role emotional, social function; Katz Index of ADLs	Mixed
1995	Health-related quality of life associated with recombinant human erythropoietin therapy for predialysis chronic renal disease patients	Revicki [50]	USA	RCT	83	Personal – Physical	Participants received HuEPO subcutaneously along with an iron supplement.	Trained staff or self-administered	Secondary	Sickness Impact Profile - home management, social interaction; SF- 36 - role function	Mixed

### Characteristics of interventions

When characterizing interventions according to the CMOP–E domains they most closely mapped onto (Table 1), most interventions exclusively addressed the Personal–Physical domain (*n = 20;* 87%). Most of the interventions within this domain used a primarily pharmacological approach (*n = 11;* 48%) or exercise-based approach (*n = 8;* 35%), with examples of interventions used including enzobiotic therapy, recombinant human erythropoietin therapy, home based exercise programs, and progressive resistance exercise training programs. The remaining three studies were characterized as Multi–Domain interventions *(n = 3;* 13%) which covered the Personal–Cognitive (*n = 3*), Personal–Affective (*n = 3*), and Environmental–Social (*n = 1*) domains. These studies used self-management interventions (*n = 2;* 9%) or other (*n = 2;* 9%) approaches. Most studies that were nonpharmacological were provided using multimodal formats with others using individual face-to-face delivery modes, (*n = 3;* 13%) and digital/e-based (*n = 1;* 4%) formats. While most studies did not report who provided the interventions (*n = 12;* 52%), reported intervention providers included trained staff (*n = 4;* 17%), self-administered (*n = 3;* 13%), exercise physiologist or physical therapist (*n = 2;* 9%), nurse (*n = 2;* 9%), principle researcher (*n = 1;* 4%), rehabilitation doctor (*n = 1;* 4%) and exercise trainer (*n = 1;* 4%) Three of the studies (13%) used multiple providers to deliver their intervention.

### Characteristics of outcomes and outcome measures

None of the 23 studies included in this review investigated life participation as a primary research outcome, and most measures used (*n = 29; 90.6%)* assessed life participation as part of a broader concept, such as quality of life or self-management behaviours. The most common life participation outcome measure used was the SF-36 (*n = 17*; 73.9%), which assessed life participation via its role physical, role emotional, and social function subscales. Other outcome measures of life participation included the KDQOL–work status, social interaction, social support, daily activities, and ADL/IADL subscales – (*n = 6;* 26%), EQ-5D – self-care and usual activity subscales – (*n = 2;* 8.7%), qualitative interviews (*n = 2;* 8.7%), Sickness Impact Profile (*n = 1;* 4.4%), Human Activity Profile (*n = 1;* 4.4%), Activities of Daily Living Scale (*n = 1;* 4.4%), Duke Activity Status Index (*n = 1;* 4.4%), Katz Index of ADLs (*n = 1;* 4.4%), and The Self-management Questionnaire (*n = 1;* 4.4%). Most of the studies (*n = 14;* 60.86%) used outcome measures that assessed all three domains of life participation (self-care, productivity and leisure), and two studies (8.7%) used outcome measures that only assessed self-care and leisure. The remainder of the studies (*n = 7;* 30.4%) did not provide sufficient information to be characterized according to the three domains. Most studies included in this review (*n = 14;* 60.9%) reported mixed life participation outcomes, with four studies (17.4%) reporting improved outcomes and five studies (21.7%) reporting no improvements.

## Discussion

In this review, we sought to identify available studies of interventions examining life participation outcomes in adults with CKD, and identify gaps in the literature. We found three key findings. First, our review underscores the lack of prioritization to investigate interventions that can promote life participation in people with CKDs. Second, we found that there has been an extremely limited breadth of intervention designs and mechanistic targets used to address life participation in CKD compared to the range of functional challenges people with CKD can face. Finally, measurement tools used in the current literature poorly capture life participation in people with CKD. Our review highlights the need for further research in this top-priority area of people with CKD to ensure they are receiving holistic and patient-focused care.

Previous research has shown that CKD is associated with declining participation in meaningful activity, and that maintaining life participation is a top priority for adults with CKD [3, 6]. In this review, we found only 23 intervention studies that assessed life participation as an outcome in this population, and no studies that examined life participation as a primary outcome. The absence of studies investigating life participation as a primary outcome demonstrate a clear lack of prioritization of this topic among nephrology researchers. This finding is consistent with what has been reported in previous explorations of similar topics; for example, a 2021 review found that lifestyle-related content was the least represented domain in self-management interventions for people with CKD, only being addressed in 11% of self-management programs [18]. The same study found that patients had only been involved in the design of one self-management program, which might help to explain the discrepancy between patient-priority research outcomes and what are actually being studied by nephrology researchers [18]. Predialysis CKD patients should have a greater say in the direction and design of future studies moving forward, so that they better align with the priorities of the population whom they are aiming to help.

Our review found that most studies (87%) used interventions that exclusively addressed the Personal–Physical domain of the CMOP-E, which includes interventions targeting movement, strength, coordination, balance, endurance, pain, appearance, and physical illness and/or injury of body systems and/or structures [22]. In particular, the most common intervention approaches studied in this review were pharmacological (47.8%) and exercise (34.8%), both physically-focused approaches. While this finding is partially explained by the fact that life participation was not the primary outcome targeted in any study, it still strongly suggests that current research relating to life participation is deeply entrenched in the medical model of care, and is overly focused on one aspect of the disability experienced by the population. This narrow focus is contrasted against evidence showing that CKD leads to cognitive changes, particularly orientation & attention and language [9]., which can interfere with life participation [12], Similarly, research has shown an impact on the psychosocial well-being of individuals with CKD, with studies finding increased rates of depression, psychological distress, and anxiety [23–26]. There is therefore a need to further investigate approaches in the CKD population, such as cognitive rehabilitation and psychotherapeutic programs, that are often used in other chronic disease groups to target nonphysical mechanisms of disability. Our review also found no studies attempting to promote life participation by targeting environmental factors, such as caregiver support, home modifications, or the use of assistive devices. Collectively, our findings show that existing research does not address the multiple, complex challenges of this patient population and their caregivers, and their need for holistic interventions that address multifaceted barriers to engaging in valued life activities.

Most of the outcome measures (90.6%) used in the studies only assessed life participation incidentally as part of a broader measure of a related or umbrella concept. For example, the most common outcome measure (73.9%), the SF-36, is a health-related quality of life measure that includes three subscales which capture life participation. As such, while most outcome measures (60.86%) in this review appeared to examine life participation comprehensively with questions addressing all three domains of self-care, work, and leisure, most did so only superficially, with one or two questions addressing each domain. We underscore limitations of using quality of life measures to assess life participation that have been acknowledged in previous reviews, such as their lack of validation in CKD populations or lack of validation as primary life participation measures. In addition, the SF-36 subscales that assess life participation have not each been investigated to establish their responsiveness to change, making their use as intervention outcome measures particularly problematic [27]. Collectively, our results indicate that studies have either not accurately captured the concept of life participation, or encapsulated enough content about life participation, to reflect the concept fully. This limitation highlights another important gap in the research literature on this topic.

The strengths of our study include the use of multiple electronic databases in our search strategy to ensure comprehensiveness. Full text review and data extraction were completed in duplicate by two independent reviewers, which is considered the gold standard for scoping reviews and presents another strength for this review. Due to the language constraints of the reviewers, this review only examined papers written in English which we acknowledge as a limitation of the study. Future research should set out to explore articles written about life participation in different languages to increase our understanding of this important topic. Moreover, life participation and occupational engagement are not well-defined terms in the literature. While our review included a broad set of terms to attempt to capture the range of published works on this topic, it is possible that other terms were used which may not have been captured in our search. It is also possible that a broader set of interventions would have been captured by including impairment/body system outcomes (e.g. gait speed, strength cognitive functioning) as eligible for the review, since these outcomes might be favoured by researchers for being more likely to demonstrate an intervention effect. However, these outcomes should not be considered proxies for life participation, since life participation is determined not only by a person’s capabilities but also by the resources, supports and skills they have to compensate for their limitations. As such, they were excluded from this review.

## Conclusion

In conclusion, this scoping review looked at papers that examined life participation in adults with CKD. The paper highlighted a lack of research and prioritization in this area, with current research using a narrow set of intervention approaches to support life participation, and few validated measures of life participation. These gaps indicate a need for further research to support this top priority health outcome for the CKD community.

## Electronic supplementary material

Below is the link to the electronic supplementary material.

Supplementary Material 1

Supplementary Material 2

## Data Availability

No datasets were generated or analysed during the current study.

## Declarations

### Author information

Co-first authors are student Occupational Therapists from the University of Toronto. The senior author is a registered Occupational Therapist and Assistant Professor from the Department of Occupational Science and Occupational Therapy at the University of Toronto. She holds a Canada Research Chair (Tier 2) in Life Participation and Kidney Disease.
