# Supplementary material for: Exploring interventions to support life participation for adults with chronic kidney disease: a scoping review
Source: BMC Nephrol. 2025 May 22;26:251. doi: 10.1186/s12882-025-04162-8 (PMC12100810; doi:10.1186/s12882-025-04162-8)
Supplement: Supplementary file 2 — Supplementary Material 2 [file 12882_2025_4162_MOESM2_ESM.pdf]

Ovid® My Account Support & Training Help Feedback Log Off Janine Farragher

Search Journals Books Multimedia My Workspace EBP Tools What's New

My Projects My Searches & Alerts My eTocs Send us your feedback

Edit Search

Search Name

Life Participation Interventions and CKD

Comment

Type your comment here

Save Cancel

Set	Search Statement	Annotations	Insert	Edit	Delete
1.	Occupation*.tw.				
2.	(Daily adj activit*).tw.				
3.	(Meaningful adj activit*).tw.				
4.	(Life adj participation).tw.				
5.	(Activit* adj of adj daily adj living).tw.				
6.	(Independent adj living).tw.				
7.	Disabilit*.tw.				
8.	(Activity adj limitation*).tw.				
9.	(Participation adj restriction*).tw.				
10.	(Daily adj function*).tw.				
11.	(Functional adj status).tw.				
12.	(Functional adj dependence).tw.				
13.	(Functional adj limitation*).tw.				
14.	(Self adj care).tw.				
15.	(Personal adj care).tw.				
16.	(Physical adj function*).tw.				
17.	(Role adj physical).tw.				
18.	(Social adj function*).tw.				
19.	(Role adj emotional).tw.				
20.	Employment.tw.				

21.	Job.tw.
22.	Leisure.tw.
23.	Recreation*.tw.
24.	Socializ*.tw.
25.	(Return adj to adj work).tw.
26.	1 or 2 or 3 or 4 or 5 or 6 or 7 or 8 or 9 or 10 or 11 or 12 or 13 or 14 or 15 or 16 or 17 or 18 or 19 or 20 or 21 or 22 or 23 or 24 or 25
27.	CKD.tw,kf.
28.	(Chronic adj Kidney adj Disease*).tw,kf.
29.	(Chronic adj Renal adj Disease*).tw,kf.
30.	(Chronic adj Kidney adj Failure).tw,kf.
31.	(Chronic adj Renal adj Failure).tw,kf.
32.	(End-Stage adj Renal adj Disease*).tw,kf.
33.	(End-Stage adj Renal adj Failure).tw,kf.
34.	(End-Stage adj Kidney adj Disease*).tw,kf.
35.	(End-Stage adj Kidney adj Failure).tw,kf.
36.	(Renal adj Failure).tw,kf.
37.	(Kidney adj Failure).tw,kf.
38.	Dialysis.tw,kf.
39.	Hemodialysis.tw,kf.
40.	Haemodialysis.tw,kf.
41.	(Renal adj Dialysis).tw,kf.
42.	(Kidney adj transplant*).tw,kf.
43.	(Renal adj transplant*).tw,kf.
44.	(Kidney adj allograft).tw,kf.
45.	(Renal adj allograft).tw,kf.
46.	(Kidney adj graft).tw,kf.
47.	(Renal adj graft).tw,kf.
48.	27 or 28 or 29 or 30 or 31 or 32 or 33 or 34 or 35 or 36 or 37 or 38 or 39 or 40 or 41 or 42 or 43 or 44 or 45 or 46 or 47
49.	26 and 48
50.	Program*.tw.
51.	Trial.tw.
52.	Therap*.tw.
53.	Rehabilitation.tw.
54.	Evaluation.tw.

55.	Experiment.tw.				
56.	50 or 51 or 52 or 53 or 54 or 55				
57.	49 and 56				
58.	exp "activities of daily living"/ or functional status/				
59.	exp Work/ or exp Return to Work/				
60.	exp Leisure Activities/				
61.	58 or 59 or 60				
62.	26 or 61				
63.	renal insufficiency, chronic/ or kidney failure, chronic/				
64.	renal replacement therapy/ or renal dialysis/ or hemodiafiltration/ or hemodialysis, home/ or peritoneal dialysis/				
65.	48 or 63 or 64				
66.	56 and 62 and 65				
67.	56 and 62 and 65				

English Français Italiano Deutsch 日本語 繁體中文 Español 简体中文 한국어

About Us Contact Us Privacy Policy Terms of Use Manage Cookie Preferences

© 2025 Ovid Technologies, Inc. All rights reserved. | OvidUI_05.13.00.014
